# Does health insurance improve utilisation of healthcare services, for chronic illnesses in Sudan?

**DOI:** 10.1186/1472-6963-14-S2-P6

**Published:** 2014-07-07

**Authors:** Isam Baloul, Maznah Dahlui

**Affiliations:** 1 Department of Social and Preventive Medicine (SPM), Faculty of Medicine, University of Malaya, Kuala Lumpur, Malaysia

## Background

Chronic illnesses require a long standing or lifelong adaptation. Health care plays a crucial role in this adaptation. Seeking health care for such conditions, in many developing countries, imposes a devastating social and economic burden to individuals and their families, in form of catastrophic health expenditures or impoverishment. Millions of chronically ill persons avoid health care use as a result. Worldwide there are promising evidences that health insurance promotes access to health care, and provides financial security. The Sudan has established an insurance scheme in 1995. The aim of this paper is to assess the impact of health insurance on access to health care, particularly for chronically-ill population. Understanding of determinants of access for this group is important to improve their use of health care.

## Methods

Data used for this study were obtained from the Sudan Health Utilisation and Expenditure Household Survey conducted in 2009 (SHUEHS 2009). The survey was conducted at nationwide covering 72,526 individuals of the 12,000 households. Chi square test and bivariate were used to describe the characteristics of people having chronic conditions. Multivariate logistic regression was performed to identify factors explaining health care utilisation for chronically ill population.

## Results

Of a sample of 72,526 Sudanese, 4608 (6.4%) had reported having chronic diseases, of them only 2351 (51%) had sought health care. Even with this low rate, disparity in use can be found with regard to socioeconomic background, insurance status, and types of the chronic diseases. While 59.4% of chronically ill insured had sought care only 48% of noninsured did so. After adjusting for all socioeconomic factors and health needs, having insurance was associated with 38% increase the likelihood of seeking care, compared to non-insured OR 1.38 (95% 1.194-1.603).

## Conclusion

From the SHUEHS 2009 data, there is evidence of regional and socioeconomic disparities in the utilisation of health care services. Fortunately, insurance membership was found to improve access to the health care services. This is important point, if the government decided to promote utilisation of health care for the chronically ill population, and manage the inequity in use of services.

